# Discrimination among American Indian and Alaska Native people: implications for public health communication

**DOI:** 10.3389/fpubh.2024.1384608

**Published:** 2024-10-22

**Authors:** Rene L. Begay, Matthew J. Roland, Irene V. Blair, Elizabeth Brondolo

**Affiliations:** ^1^Centers for American Indian and Alaska Native Health, University of Colorado Anschutz Medical Campus, Aurora, CO, United States; ^2^Department of Psychology, St. John’s University, Jamaica, NY, United States; ^3^Department of Psychology and Neuroscience, University of Colorado Boulder, Boulder, CO, United States

**Keywords:** Native American, American Indian and Alaska Native, Indigenous People, discrimination, microaggression, prejudice, resilience, mixed-method approach

## Abstract

**Introduction:**

American Indian and Alaska Native People (AI/AN) have experienced discrimination stemming from sustained attempts to erase AI/AN People and their culture or livelihood. Research identifying the types of discrimination experienced by AI/AN People is needed to help individuals recognize discrimination in daily life. We examine experiences of discrimination among an urban AI/AN population using a mixed methods approach.

**Methods:**

Self-identified AI/AN participants (*N* = 303, *n* = 294 with complete data; 63% women, mean age = 43 years) were recruited from the Denver-metro area in Colorado. Stress and coping models of discrimination guided our analysis. Exposure to discrimination was quantitatively assessed via the Brief Perceived Ethnic Questionnaire – Community Version (BPEDQ-CV), a self-report measure including four subscales assessing workplace discrimination, social exclusion, physical threat and harassment, and stigmatization. Participants responded to a laboratory recall task in which they described an episode of discrimination and their affective and coping responses. Content analysis was conducted on transcribed responses to illustrate discrimination exposure as reported in the BPEDQ-CV and in prior theoretical work on coping with discrimination.

**Results:**

Repeated measures analyses revealed participants reported experiencing social exclusion more than other forms of discrimination, followed by reports of workplace discrimination, stigmatization, and physical threat. Consistent with these quantitative findings, participants were more likely to recall experiences of social threat (94%), including episodes of workplace discrimination, social exclusion, and stigmatization than physical threat and harassment. Almost half the participants (47%) reported confronting or directly addressing the discrimination, and 38% reported avoiding a direct approach. For 44% of participants, their predominant emotional response included internalizing emotions such as fear/sadness/embarrassment, and another 44% reported experiencing externalizing emotions, including anger.

**Conclusion:**

Our descriptive findings present the experiences of urban AI/AN People who have experienced many forms of unjust and prejudicial treatment. These data can provide useful information to help the general public and AI/AN individuals more readily recognize and prevent discriminatory behavior, and consequently mitigate deleterious effects of discrimination on health.

## 1 Introduction

American Indian or Alaska Native (AI/AN) People have been persistently exposed to high levels of structural discrimination, including cultural and institutional threats (1). AI/AN People have faced systematic attempts to eradicate their identity and culture, including forced removal from their homes and placement in boarding schools whose purpose was to remove attachment to their culture (2, 3). At the individual level, AI/AN People have been exposed to a wide variety of interpersonal discrimination, defined as interpersonal maltreatment motivated by racial or ethnic bias (4–6).

Data on the prevalence of discrimination exposure among AI/AN People living on tribal lands or in urban communities is still limited. The findings suggest rates of discrimination against AI/AN populations are among the highest across ethnic/racial groups in the United States (U.S.) (2, 11), with more than half of AI/AN People reporting exposure to discrimination (2, 7–10). For example, findings on the prevalence of interpersonal discrimination targeted toward reservation-based AI/AN children and adolescents reveal that almost half of the participants reported facing discrimination at some point in their lives (8). Adult AI/AN participants were more likely than African American or White participants to report discrimination by healthcare professionals (11). Other studies report that a large proportion of participants experienced discrimination from employers (31%), police (32%), and healthcare institutions (23%) (12).

Racial and ethnic discrimination has been recognized as a public health threat by the American Medical Association (13). To address this threat among AI/AN People, there is a need for broader public awareness of the specific types of prejudice and discrimination AI/AN individuals face. This knowledge may guide the development of public health interventions to decrease discrimination by promoting increased awareness of common types of prejudicial communications and discriminatory behavior. Information presented in an accessible and relatable manner could heighten non-AI/AN individuals’ awareness of words and actions that communicate prejudice, potentially reducing discriminatory behavior.

Knowledge about discriminatory acts may also protect AI/AN People from harms associated with discrimination. This information may help targeted Indigenous individuals more quickly recognize prejudicial and potentially discriminatory behavior, potentially reducing the stress evoked as individuals try to evaluate subtle forms of discrimination (14). Therefore, the present study aims to link findings from theoretical and empirical studies of discrimination with descriptions of lived experiences of discrimination and associated coping strategies reported by AI/AN People.

Key dimensions of interpersonal discrimination include a broad variety of maltreatment including social exclusion, unfair treatment at school or work, social and physical harassment, and stigmatization (15, 16). These discriminatory threats emerge from stereotypes about AI/AN People, including beliefs about their associations to substance abuse or criminal acts (17). Other discriminatory behavior reflects the traumatic history of AI/AN Peoples in the U.S. For example, AI/AN culture can be rendered invisible when members of other groups believe the AI/AN People only existed in the past and that none survived to the present day. When individuals are unaware of AI/AN communities, they may deny that an individual is Indigenous because they fail to recognize AI/AN People as a group whose members have unique languages, ways of knowing, educational pursuits, families, jobs, and certain cultural practices (e.g., wearing ceremonial apparel) (17, 18).

A limited body of quantitative research has examined the types of discrimination that AI/AN individuals face. Blair et al. (2) examined exposure to different types of interpersonal discrimination among a convenience sample of urban dwelling AI/AN People using the Brief Perceived Ethnic Discrimination -Community Version (Brief PEDQ-CV). The Brief-PEDQ-CV emerged from studies of ethnicity-related stress by Contrada et al. (16, 19) and Brondolo et al. (15). The Brief PEDQ-CV permits examination of different types of discrimination including racebased episodes of social threats (i.e., social exclusion, stigmatization and work or school-based discrimination) as well as physical threats. Findings on the types of discrimination reported by AI/AN People were compared to data from convenience samples of urban African American and Asian individuals recruited from communities in New York City (2). For AI/AN People, as is the case with other groups, social exclusion was the most prevalent form of discrimination, followed by workplace discrimination and stigmatization. Physical threat was the least common exposure.

These data are consistent with other studies examining variations in exposure to different types of discrimination. Among adolescent participants, studies have suggested that verbal harassment is the most common type of discriminatory behavior expressed by outgroups (7). Verbal harassment can include slurs, insults, verbal threats, criticism, and harmful exclusion. A recent report by D’Amico et al. (7) documented similar trends, reporting that a sizable percentage of participants reported being asked to prove the authenticity of their AI/AN status, consistent with the notion that invisibility is a salient form of discriminatory behavior.

Although participants report that race-based physical maltreatment is the least common type of maltreatment, physical threats remain a serious issue. A recent study reported that a quarter of adult participants in their sample indicated that they or their family members were subject to violence and threats (12). Studies including adolescents indicated that 14% reported exposure to physical threat (7).

Qualitative data are needed to facilitate the recognition of and understanding of these different types of discrimination. Qualitative data can provide insight into the lived experiences of discrimination in everyday life as they are drawn from personalistic accounts from individuals within the groups being studied (17, 20–22). As Robertson (17) noted, qualitative methodology allows researchers to, “[give] value and voice to their lived experiences in a historically contextualized way.”

Existing qualitative studies have examined perceptions of the link between discrimination and specific issues of importance to the AI/AN community, including substance use (21, 23), health trajectories (6, 24), academic achievement (25), and resiliency in the face of discrimination (26–29). For instance, some participants in the Skewes and Blume (21) paper identified discrimination as an underlying cause or perpetuator of their substance use. One participant even stated that, “Oppression is the overarching umbrella for all sickness with drugs and alcohol,” (21). Solomon et al. (24) and Brondolo et al. (45) documented barriers associated with the Coronavirus Disease 2019 (COVID-19) pandemic that created health disparities in treatment and administration of vaccines. Other studies have provided insights into the ways in which a focus on tradition and interconnectedness among American Indian communities can build resilience against discrimination (28, 29).

However, there are gaps in knowledge. To date, existing studies of discrimination among AI/AN People have not explicitly tied descriptions of the lived experiences of discrimination to models of interpersonal discrimination derived from quantitative analyses, such as those of Contrada et al. (16, 19) or Krieger (4). These models describe distinctions among different types of discriminatory experiences. Analyses linking model-based quantitative data to qualitative findings could provide an accessible roadmap to understanding how to conceptualize discriminatory experiences and recognize them in different contexts. Combining quantitative and qualitative data can support Skewes and Blume’s (21) call for educational interventions to reduce the discrimination AI/AN People have experienced and provide guidance for the development of public health interventions to help mitigate resulting health issues.

In the present study, we combined both quantitative and qualitative approaches to provide insight into discrimination exposure among urban AI/AN People. In contrast to qualitative studies of AI/AN People that incorporated inductive, grounded theory-based approaches that extrapolate themes (17, 18, 20, 21, 26), we employed another qualitative approach, content analysis, to provide illustrations of lived experiences of race-related discriminatory experiences, including social threat, stigmatization, work or school-based unfair treatment, and physical harassment. Content analysis was used to depict illustrations of specific aspects of extant theoretical frameworks of discrimination. Excerpts were analyzed to determine which types of discrimination and coping were identified.

In addition to illustrating experiences of discrimination, we expand the existing qualitative literature to include theoretically derived assessments of coping. Using frameworks developed by Krieger (27) and Krieger (4), coping responses have been categorized as either avoiding addressing the maltreatment or directly confronting the maltreatment. Approaches which focus on suppression or expression are also consistent with other research on the use of anger-coping strategies (i.e., anger-in vs. anger-out) in studies of coping with racism by Black and Hispanic Americans (46). We assess resilience as some researchers have reported that exposure to trauma (such as discrimination) can build resilience through the development of adaptive coping strategies, including seeking out social support (30).

## 2 Methods

### 2.1 Study population

The total sample population in this study consisted of 303 urban AI/AN People living in the Denver-metro area in Colorado. Nine participants chose to exit the study due to concerns raised during the screening tests (e.g., severely elevated blood pressure) or they did not attend study testing sessions, leaving 294 participants as the study sample.

In the U.S., AI/AN People make up roughly 2% of the population. They often identify either with one of the 575 federally- recognized or state-recognized tribes or with those that are unrecognized. Federally recognized tribes span 35 states with a large concentration located in the southwest region.

Roughly 46,000 AI/AN People live in the Denver-metro area alone (47). Denver has a growing population of urban AI/AN People because it sits between the Southwest and Midwest plains regions where many Tribal lands are located (47). Estimates suggest that 78% of AI/AN People live in urban areas; however, this number may fluctuate as many people are tied to their community, land, and relatives/family that are located on Tribal lands, but travel into urban areas for education, healthcare, or work (48). Over half (53%) of the participants in this study were affiliated with tribes from the Great Plains region of the U.S. while second largest group were affiliated with a tribe located in the Southwest (11%). Several participants noted more than one affiliation (20%). Forty participants did not respond with their tribal affiliation.

To participate in this study, we required participants to self-identify as an AI/AN individual, aged 18 or older, and residing in the Denver metro area. Participants were excluded if they had a pacemaker, were pregnant, were on dialysis, unable to have blood pressure measured, frequently used illicit drugs, were unable to provide consent, or who had severely elevated blood pressure. The study sample was comprised of 63.3% women (*n* = 186), and 36.7% men (*n* = 108), with a mean age of 43.4 (SD = 14.7, Range: 18–78 years). The majority (*n* = 171; 62.1%) were living at or below the poverty level, and 52 (11%) were living at three times or more than the poverty level; 47.6% (*n* = 140) had completed some college or more education and 52.4% (*n* = 154) had a high school diploma or less; and 38.2% (*n* = 112) were married.

### 2.2 Recruitment

Participants were recruited via word of mouth and via flyers that were sent electronically and posted physically inside the local Tribal community centers, health clinic, and public bulletin boards and at annual Tribal events. Participants could reach out to the study team by phone call or email to discuss their eligibility and to schedule a visit to the University clinic. Once contact was made, the participant was mailed a consent form detailing the benefits and limitations of the study before their first visit. Before their first visit, the participant was once again given details of the study and consent was obtained following a discussion to ensure voluntary and informed consent.

### 2.3 Data collection and mixed methods analyses

Urban AI/AN individuals participated in a field and laboratory study on stress and health that was funded by the American Heart Association. Informed consent was conducted in-person and privately at the research office. The research assistant reviewed all study procedures with each participant, using printed visual aids to depict the flow of the study session. Consent was provided in writing and participants were given a copy of the document. As a part of the first laboratory session, participants completed a quantitative measure of perceived ethnic discrimination and orally responded to a prompt to think about experiences of discrimination to describe, how they felt, and to detail the ways they coped with these experiences. Further details of the study have been previously published (31), and are publicly available at: http://tinyurl.com/5wbsc9ad. To gather information about the participants’ experiences of discrimination, the interviewers provided the participants with a tape recorder in their room. The tape recorder was then turned on by the participant when a pre-recording prompt said, “Talk about a past experience in which you knew you were treated poorly or unfairly because you are American Indian, even if the other person did not mention your AI/AN cultural status. We will record what you say. We are not able to talk to you about this experience until your return visit the next day.” Participants were also provided with the following six pilot tested written prompts to help them detail the act of discrimination that they planned to discuss: “What happened?,” “Who were you with?,” “Where were you?,” “What did the other person do or say?,” “How did you feel?,” and “What did you do or say?.” To answer these prompts, the participants were given 2 minutes to prepare, and then given the opportunity to discuss their discriminatory experience with no time limit. The response time per participant was 2.2 minutes on average. Participant responses to these prompts were recorded and transcribed and served as the data for the qualitative portion of the study.

The laboratory protocol in which participants provided descriptions of these experiences were administered and collected by research assistants in a clinic at the University of Colorado Boulder from 2016 to 2019.

### 2.4 Statement of positionality

The principal investigators of the study are an Asian American gender-queer woman (IB) and a White woman (EB). The Indigenous woman co-lead author (RB) served as a community advisory board member and is also a public health researcher who saw the value of the project. The research team asked her to lead this project and she was willing to help curate the message behind this paper. Her lived experiences as an Indigenous (Diné/Navajo), living in rural/urban settings provided a necessary and unique lens. The other co-lead author (MR), a White man, is a student who has spent several years working in the Collaborative Health Integration Research Program (CHIRP, directed by EB), a research training program focused on health disparities. He contributed to the analyses and organization of the data for the project which provided the structure needed to conclude the data. EB is a clinical psychologist who has devoted her research career to studying minority health disparities (45, 46). She met with the research assistants and coders each week to help them process the emotional impact of the stories that were conveyed. These discussions provided opportunities for her to learn about the types of discrimination the research participants reported and to understand the effect hearing these stories had on others. IB is a social psychologist who has dedicated her research career to understanding and addressing prejudice, racism, and health disparities. IB acknowledges her positionality as a descendant of people who came to this country as colonial/settlers and immigrants. The research assistants involved in the qualitative coding process included one White man and five men and women from underrepresented groups (e.g., Black, Latino/a, and Asian American). The research assistants received intensive training and supervision in explaining the study, administering the laboratory protocols, and training on the questions about discrimination. Weekly review sessions were held to support interviewers and ensure adherence to the testing protocol.

### 2.5 Measures

Quantitative assessment of exposure to racial discrimination was obtained with the Brief Perceived Ethnic Questionnaire – Community Version [BPEDQ-CV (15)]. The BPEDQ-CV is a short 17-item assessment of lifetime exposure to discrimination derived from the 34-item Perceived Ethnic Discrimination Questionnaire – Community Version [PEDQ-CV; (15)]. The BPEDQ-CV includes four scales that assess different dimensions of discrimination, including: exclusion/rejection, stigmatization/devaluation, discrimination at work/school, and physical threat/aggression. Items are scored on a Likert scale from 1 (“Never happened”) to 5 (“Happened very often”). Some example items included: “Have you been treated unfairly by teachers, principals, or other staff at school,” “Have others thought you could not do things or handle a job,” and “Have others threatened to hurt you (ex: said they would hit you)?” A mean score was calculated such that higher scores indicated greater exposure to discrimination (time 1 *α* = 0.90). Findings from our prior studies comparing new data from AI/AN individuals to data drawn from samples of urban African American and Asian individuals tested in similar circumstances in New York City, New York (2) which indicated the Brief PEDQ-CV demonstrated measurement invariance across all three urban groups. This suggests the Brief PEDQ-CV is a suitable tool for use with the AI/AN sample population. The Brief PEDQ-CV was administered on the first visit. A research assistant read the questions aloud to the participants and recorded their responses in RedCap.

### 2.6 Content analysis

Interviews were transcribed verbatim and were analyzed by six coders from St. John’s University (three groups of two). The six coders included three men and three women who were all undergraduate students. The coders were trained by the research assistants at the University of Colorado Boulder, who developed the protocols, and by the principal investigators. Dedoose software was used to organize, manage the data, and apply the codes to the transcripts. Before beginning coding, Dedoose software calculated the Kappa statistic (inter-rater agreement) achieved when coding a pre-selected set of interview excerpts. Coding began once raters achieved Kappa levels above 0.60. Actual Kappa levels ranged from 0.68 to 0.87. Obtaining these kappa ranges involved an iterative process of refining codes and re-testing until sufficient agreement was reached among the coders.

Codes were generated *a priori* to reflect the types of discrimination and responses to discrimination based on prior work on racial and ethnic discrimination (15) and specific studies of discrimination facing AI/AN People (2, 31, 32). The codes and their explicit definitions were developed by researchers and research assistants (See Supplementary Tables S1–S7). A total of 41 codes listed under seven broader topics (See Supplementary Table S8) were created describing aspects of the participants’ experiences of discrimination. In this paper we analyzed codes related to three topics (See Supplementary Tables S8, S9): (1) the act of discrimination (coding for different types of discrimination), (2) affective and behavioral responses to the act of discrimination (e.g., sad, frustrated, angry, confrontation, or avoidance etc.), (3) and evidence of resilience. Chi-Square analyses were performed to determine significant differences in frequency among related categories.

### 2.7 Ethics

This study was approved by St. John’s University Institutional Review Board (IRB), University of Colorado Boulder IRB, and the Colorado Multiple Institutional Review Board (COMIRB). Feedback on the protocol, implementation, and interpretation of the results were provided by the community advisory board (CAB) composed of an all Indigenous (AI/AN) panel composed of six individuals living in the Denver-metro area. The CAB met with varying frequency over the course of several years, approximately 2–3 times a year. CAB members were initially recruited with the assistance of an AI/AN community liaison employed by the Centers for American Indian and Alaska Native Health (CAIANH). The goal was to include individuals who identify as AI/AN with a range of ages and gender, who are involved in the community and knowledgeable about AI/AN cultures and perspectives represented in the Denver-metro area. Board members were compensated for their time and expertise. All participants whose excerpts are cited in this paper provided written permission for their excerpts to be included and published.

## 3 Results

### 3.1 Quantitative analyses

**Figure 1:**
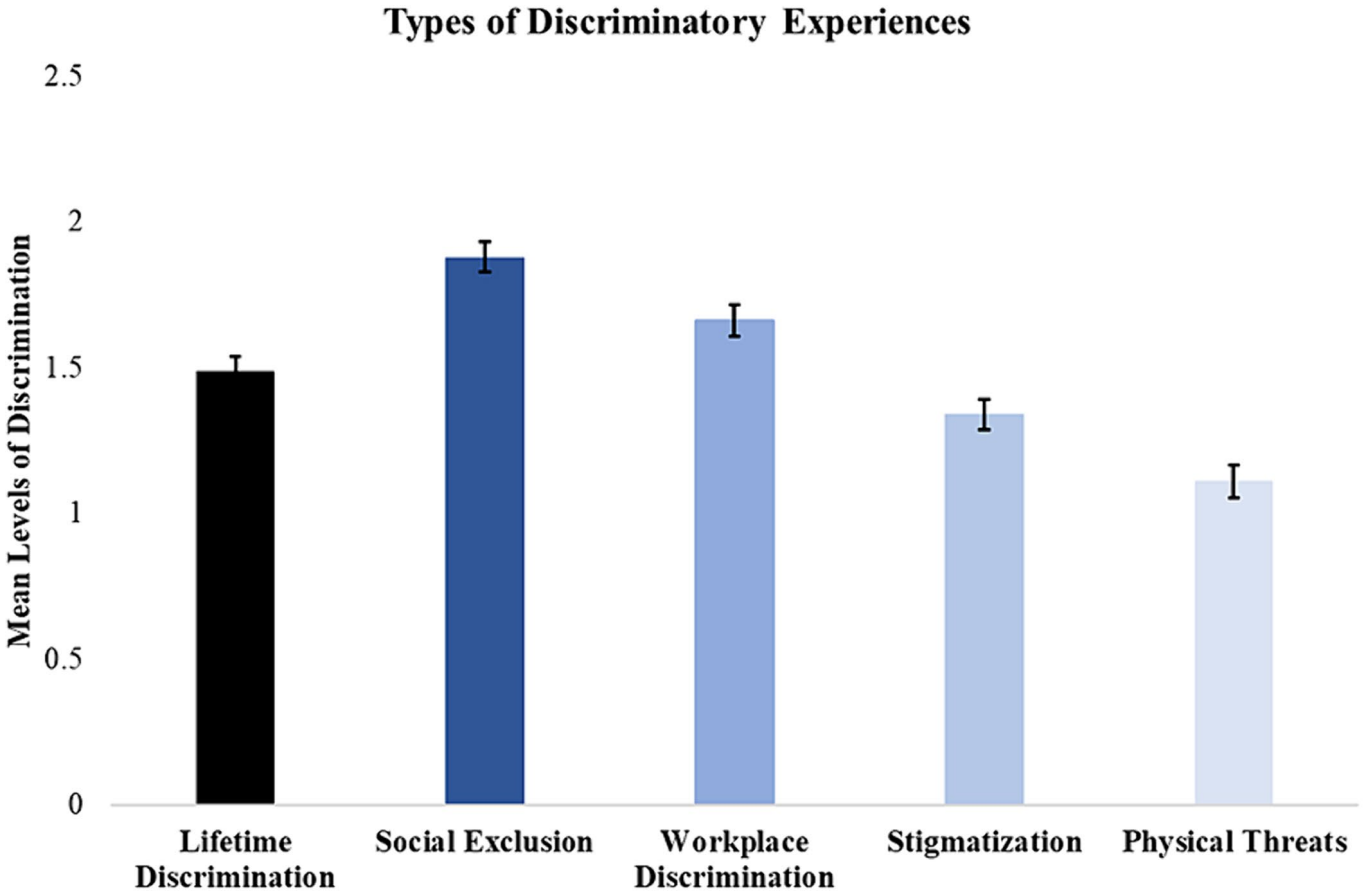
Mean rates of discrimination subtypes measured by the BPEDQ-CV faced by our sample (*n* = 294). Survey data suggest that social exclusion was the most common type of discriminatory experience (*M* = 1.88; SEM = 0.05), followed by workplace-based discrimination (*M* = 1.66; SEM = 0.05), stigmatization (*M* = 1.34; SEM = 0.05), and physical threats of violence (*M* = 1.11; SEM = 0.06).

**Figure 2:**
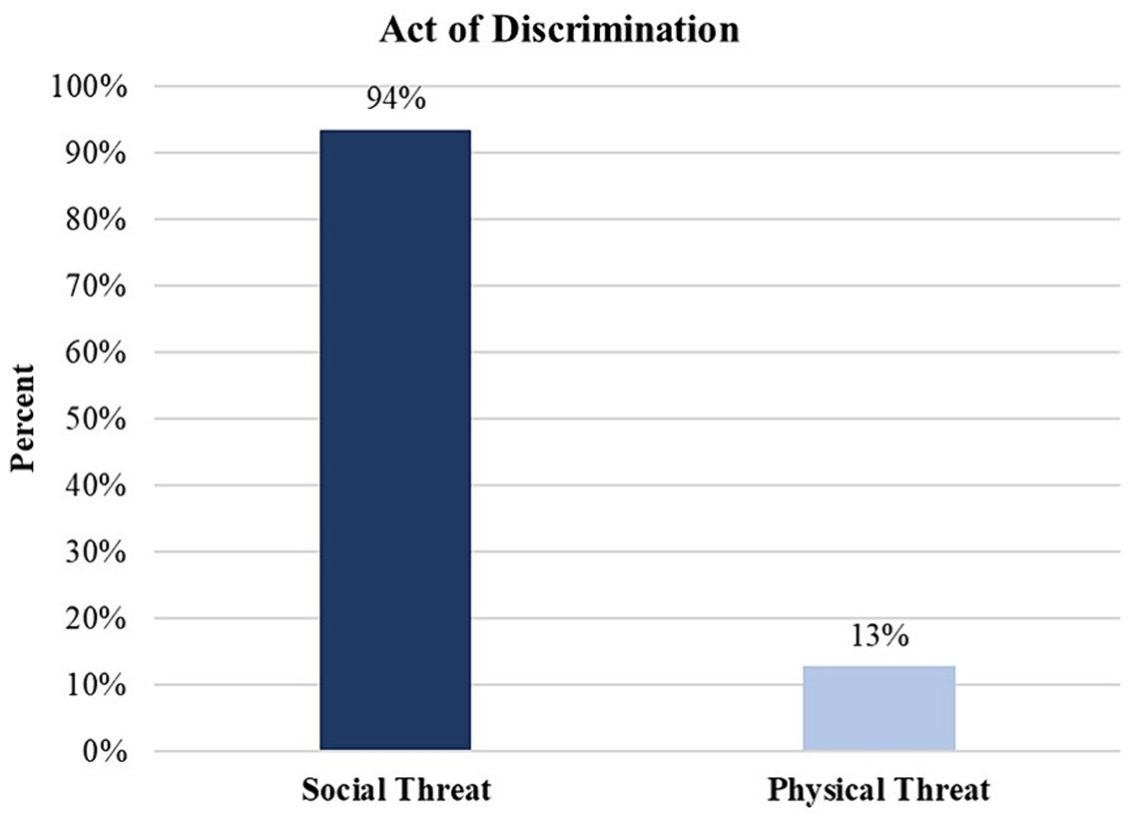
Act of discrimination experienced by AI/AN participants. Percentages represent the number of times the type of threat occurred across all participant excerpts divided by the total number of study participants (*n* = 294). As a consequence, they are not mutually exclusive codes. Some participants reported both social threats and physical threats, or not at all. Thus, the percentages presented in this figure exceed 100%. A chi-square goodness of fit test was performed to assess differences in the frequency of acts of discrimination, *Χ*^2^(1) = 179.45, *p* < 0.0001. Results suggest that social threats are the most prevalent act of discrimination, more common than physical threat.

**Figure 3:**
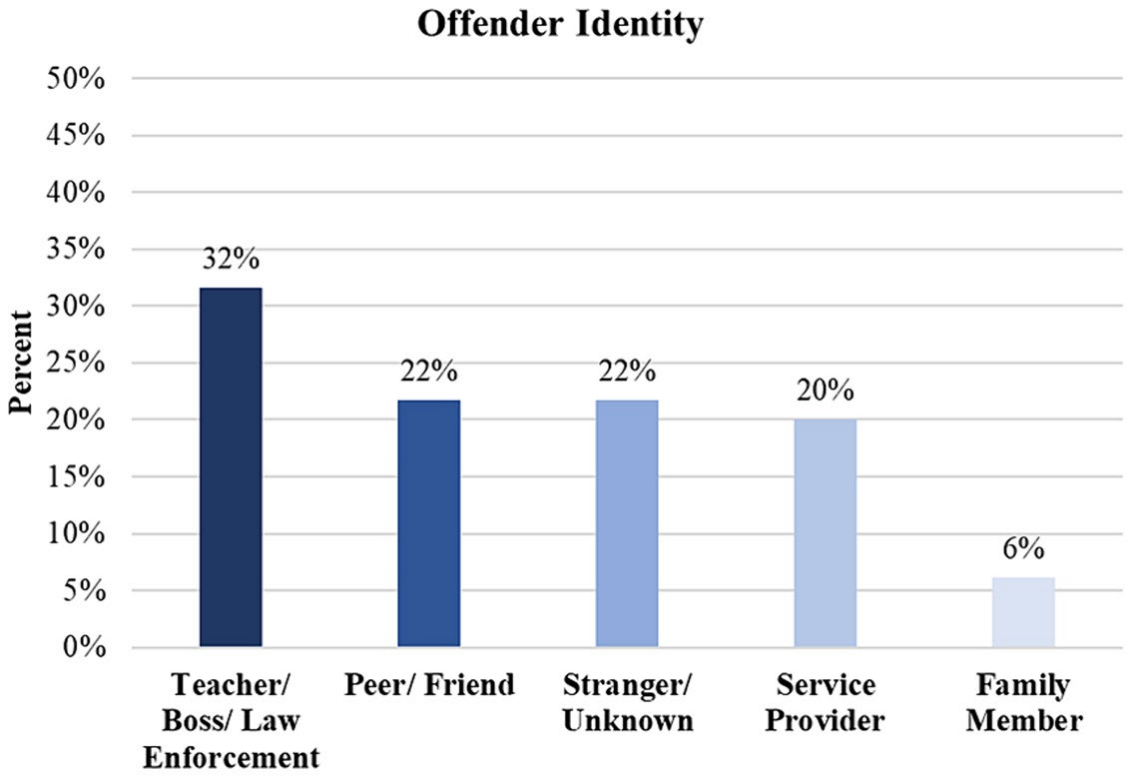
General identity of offender who committed a discriminatory act. Percentages represent the figures of authority of the offender identities divided by the total number of study participants (*n* = 294). As such, multiple types of offenders may have appeared in certain participants’ excerpts. Thus, the percentages presented in this figure exceed 100%. A chi-square goodness of fit test revealed significant differences in observed frequencies among the offender subtypes, *Χ*^2^(4) = 48.41, *p* < 0.0001. These findings suggest that authority figures, such as teachers, bosses, and law enforcement figures are more commonly perceived as perpetrators compared to other offender subtypes.

**Figure 4:**
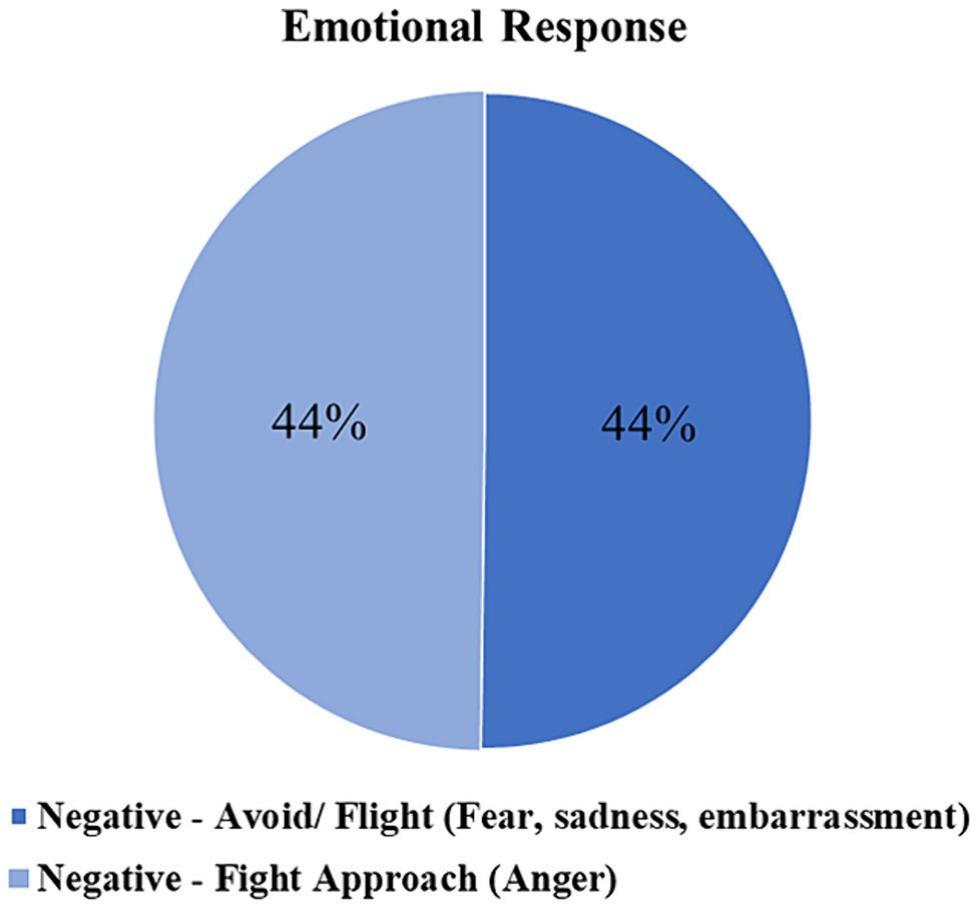
Emotional response by participants. Percentages represent the number of negative emotional flight approach (or fight approach) responses divided by the total number of study participants (*n* = 294). As a consequence, the types of emotional responses are not mutually exclusive, and some emotional responses may not have been recorded. Thus, the percentages presented in this figure do not sum up to 100%. A chi-square goodness of fit test was performed to assess differences in observed frequencies of emotional responses, revealing non-significant differences, *Χ*^2^(1) = 0.004, *p* > 0.05. These findings indicate that anger and avoidance emotional responses are equally common responses to discriminatory behavior.

Linear mixed models indicate that mean scores across the four scales significantly differed from each other (F(1,293) = 138.06, *p* <0 .001). Consistent with previous studies (7, 8), our findings suggest that social exclusion (*M* = 1.89; SE = 0.05) is the most prevalent type of discrimination, followed by workplace-based discrimination (*M* = 1.66; SE = 0.05), stigmatization (*M* = 1.34; SE = 0.05), and physical threats of violence (*M* = 1.11; SE = 0.06) (see Figure 1). Results of quantitative analyses of the qualitative data are presented in Figures 2–4.

### 3.2 Qualitative analyses

Responses were coded as acts of discrimination if the participant described specific acts of maltreatment related to their AI/AN status. We organized responses into the type of maltreatment including episodes of social exclusion, unfair workplace/school treatment, stigmatization (i.e., being called racial slurs or hearing references to negative stereotypes), and physical maltreatment. In addition to episodes of social exclusion that involved being ignored or left out, we included denial of the existence of AI/AN People or denial of an individual’s Indigeneity as a specific form of social exclusion, as the individual was excluded both from the mainstream culture and their Indigenous culture to which they belonged. Figure 2 also details the frequency with which categories of “Act of Discrimination” codes were employed. To illustrate these categories, we included excerpts drawn from the participants’ response to the laboratory discrimination-recall task which provide examples of specific types of discrimination identified. It is important to note that some episodes reflect more than one type of discrimination. In such cases, these additional types are noted at the end of the excerpt. Detailed explanations of the characteristics associated with each code are provided in the Supplementary Tables S1–S7. The second topic included participant’s “Responses to Discrimination.” These responses were further organized into affective responses and then coping responses which could include episodes of avoidance and suppression, confrontation or directly addressing discrimination. The third topic was “Resilience” in the face of discrimination.

Lastly, we highlighted the groups who were the aggressors or offenders toward the AI/AN individuals. We found that individuals with authority (32%) (e.g., teachers, bosses, or law enforcement officials) were reported most frequently as committing an act of discrimination against the target individual, followed by peers and friends (22%), strangers (22%), service providers (20%) (e.g., store or restaurant employees, medical staff, etc.), and family members (6%) (see Figure 3).

### 3.3 A: Acts of discrimination

1: Descriptions of Social Exclusion

These lived experience descriptions of social exclusion include experiences of being rejected, ignored, and isolated (excerpt A.1.1) and ostracized (excerpt A.1.2). These episodes also include examples of being told AI/AN culture does not exist or that one is not AI/AN (excerpts A.1.3–A.1.7).

Excerpt A.1.1 “As a child, I got information about my father from my grandma, and through the story she told me his ethnicity and everything. As a child, I did not really feel a part. I felt kind of isolated a lot, so it kind of gave, I guess, understanding to me why I felt so isolated and different, aside from my mom, brother, her husband. Like, they were like a family and it seemed like I was separate, and finding out that my dad was Native…So it gave me something to hold on to. Just going through the growing up stage, there was a lot of riding in a car with your head down between your legs so you do not get a chance to see -- you are not seen in the car as a part of the family riding in the car…Like, if she goes to visit -- or take my brother to visit his father, I would have to stay in the car. Like I wasn’t accepted.” (Man, 30–40).

A.1.2 “A few years ago, I went back home to visit some family members. Got a hotel room and invited my family to come over and swim, all the little relatives. And while we were swimming, a Caucasian woman came up to me and asked me how long we were planning on being in the pool. I asked her, well, why is that any of her business? She, then, told me that she did not want her kids in the same pool as us Native Americans. That we were swimming for more than two hours, and we had enough time to be in the pool, and we should leave because her kids needed to go swimming (Woman, 20–30). Note: This example could also be considered stigmatization if the perpetrator was implying that AI/AN children were unclean.

A.1.3. “I was at…a nonprofit place and I had to fill out some paperwork…When I got to their forms where you fill out your ethnicity, race, all that good stuff, I actually wrote down Native and human, and the lady came back up to me and she said, “How are you Native American? You’re too dark.”…it’s not the first time that I mentioned being native and someone kind of just brushes it off like, no you are not, you do not fit the profile of what they ‘look like.’” (Woman, 20–30).

A.1.4 “Well, I used to pass by a Native American cultural center on [Street Name], and one day I wanted to go in just to see what it was about… everybody was different from me. They had braids, long hair, and they looked at me, and they asked me, “What do you want? What are you doing here?” I said, “I want to learn about my people, my tribe.” He said, “But, you are not Native American.” (Man, 50–60).

A.1.5. “When I was in high school for my senior year… there is Native American little classes and stuff where we can go and talk about us, what our ethnicity was and just share our stories…But this one boy and his sister… said that the brother would always get bullied because he had long hair and he would always come with a braid and everything in his hair and everybody thought he was a girl because he had long hair. That touched me because all my family has the long hair and everything. A lot of people just be like “Oh, he’s a girl. He wants to have long hair and everything.” (Woman, 18–28).

A.1.6 “When I was growing up, I was constantly teased and bullied over having long hair. They always made fun of me all the time. I was with my little brother, happened with him too, usually at school. The other people used to make homophobic comments and say things, implying that I was homosexual because I had long hair.” (Male, 30–40).

A.1.7. “During the president’s State of the Union address, he illustrated unemployment for the blacks, for the whites, for the Latinos, but never mentioned the first Americans. Many cases where the government has failed to address Indian issues, such as the rights of the pipelines up north. That was a heart-wrenching one for us natives. Any time we do something, it’s always about not being recognized as an existing group of people…What about Native Americans? We’re forgotten. Our rights do not count no more because we are first Americans.” (Man, 60–70).

2: Descriptions of Workplace or School Discrimination

These lived experience descriptions of workplace or school discrimination includes episodes in which individuals describe being assigned tasks (see excerpt A.2.1) or not assigned tasks (see excerpt A.2.2) as a function of biased assumptions about their capabilities. These biased assumptions may be driven by stereotypes about AI/AN People.

A.2.1 “[The participant was at work and her manager assumed she was Latina and asked her to translate for a Spanish speaking customer] A coworker said “……. you could probably translate for us.’ I looked at her, and then I said, ‘What makes you think that?’ She said, ‘Well, your last name is [Participant’s Last Name], is not it?’ I told her, ‘That does not have to do with my ethnicity. I’m not even Mexican or Hispanic at all.’ She just looked at me with a blank stare, and then she told me, “Well, what are you?” I told her, ‘I’m Native American, hundred percent…’ She was just like, ‘Oh, okay then. I just thought [Participant’s Last Name] came from a Hispanic heritage.’ I just told her, ‘You’re not the first. Everyone assumes that. You should not judge a book by its cover, though.’ (Woman, 20–30) Note: Example A.2.1 could also be considered a form of social exclusion involving the denial of one’s ethnicity.

A.2.2 “My experience occurred when I was at work. What happened was a patient’s family member would not allow me to care for them because another co-worker had mentioned that I was Native American, and so I was asked to leave the room.” (Woman, 20–30).

A.2.3 “When I told [my teacher] I wanted to be in the military and wanted to do these things, he told me I could not, saying that my writing skills and my speech and my way I carried myself, he said it was -- I would, it will never happen by the way I, by the way I was supposedly by the way my grades were. So, and I felt really bad about that; it made me feel, it made me feel like I could not do anything after that when he told me that.” (Man, 20–30).

3: Descriptions of Stigmatization

Some of these lived experience descriptions expressions of stigmatization include the invocation or assignment of negative stereotypes about the group (Excerpt A.3.1). Other expressions of stigmatization include verbal harassment, including slurs, of the targeted individuals (Excerpts A.3.2 – A.3.4).

A.3.1 “I was at work. I was filling out my timesheet. Getting ready to go home. When this Caucasian gentleman he walked up to me, my supervisor at that, and he said to me, he asked me, he said, “What are you doing? Are you signing over the deed to the reservation?” (Man, 40–50). Note: Example could also be considered workplace discrimination.

A.3.2 “I was at [Name] Middle School [State/City] with my friend, ***, who was also [Tribe Name]. We were outside after lunch. We were approached by two white girls who began to call us names. They called us “dirty Indians” and other derogatory names.” (Woman, 40–50).

A.3.3 “…just hanging out with everybody it would just get brought up from time to time that I was Native American and they would tease me about just by slapping their mouth and making, like, the hollering noise that’s notorious for being done.” (Man, 20–30).

A.3.4 They would ask me if I smoke tobacco or if I’m alcoholic, or we’d occasionally talk about the Reservations and they would kind of tease me about that from time to time.” (Man, 20–30).

4: Descriptions of Physical Threat

These lived experience descriptions of physical threat and harassment include implied or actual physical assault on the participant. These episodes are often accompanied by verbal maltreatment but are distinguished by menacing behavior, including the threat of physical violence (Excerpt A.4.1) or actual violence (A.4.2 and A.4.3). Quantitative data indicates that physical harassment scores on the Brief PEDQ-CV were lower than scores on measures involving social exclusion, stigmatization, and workplace discrimination. This pattern is consistent with the qualitative data in which a smaller proportion (13%) of individuals reported on an event in which they faced discrimination-related physical harassment. Examples of physical threats or harassment are illustrated below.

A.4.1 “…we went for supplies to a nearby town to shop…one of my friends got spit in the face and told to go home. We were being followed by police. We were being followed by locals, who were -- wanted us to leave. They would tell us to go home, call us nasty names, whatever -- even the hotels, they would accost us.” (Woman, 40–50).

A.4.2 “I got attacked by a dog. I was with a white friend. I was at the park. The other person was a white guy. He had a dog and he let it loose and he was like “red meat.” (Man, 40–50).

A.4.3 “So this was a incident when I was about 13 years old. I got in a fight at school. It’s a fist fight with a guy over a girl, and the police were called. And when they got there, I had a gun pointed in my face. I got hit in the back of the head with the gun, and this is just for getting into a fight. And the cop told me – I felt it was because it was my race and stuff – that I was – he was going to make sure I spent the rest of my life in jail.” (Man, 40–50).

### 3.4 B: Responses to discrimination

These lived experience descriptions were used to classify emotional responses. Codes about emotions were applied if the participant identified the emotion they experienced following the discriminatory act. We categorized these emotions into two dimensions: anger-related emotions (e.g., including reports of feeling angry, mad, hostile, and related terms) and fear/sadness-related emotions (e.g., including reports of feeling sad, upset, afraid, anxious). This distinction between anger-related and fear/sadness related dimensions is common in the clinical literature (33). “First Party (Speaker) Response/ Intent” codes were applied to responses when participants were asked about their emotional responses to the situation and their coping responses to the situation and their own emotions. We found that 44% of the participants felt an anger-related emotion compared to 44% of participants who felt sadness or anxiety, including feeling fearful or upset in response to the act of discrimination. Figure 4 presents the frequency of “Emotional Response.”

**Figure 5:**
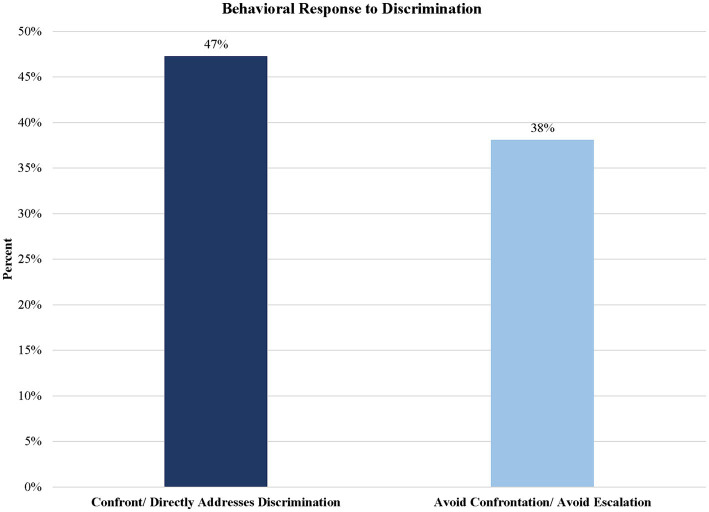
Individuals who confronted or avoided discrimination from others. Percentages represent the number of confrontations (or avoidance) divided by the total number of study participants (*n* = 294). As a consequence, some of the confrontation (or avoidance) responses were not mutually exclusive for some participants, or they may not have appeared at all for others. Thus, the percentages presented in this figure do not sum up to 100%. A chi-square goodness of fit test was performed to assess differences in observed frequencies of confrontational or avoidance behaviors, revealing non-significant differences, *Χ*^2^(1) = 2.90, *p* > 0.05. These findings suggest that confrontational and avoidance behaviors are equally common responses to discriminatory behavior.

The next set of codes were used to classify coping responses. Two major categories were identified based on the literature (34): “Avoiding Confrontation/Emotional Suppression” and “Confront Directly/Addresses Discrimination.” Responses were defined as “Confront/Directly Addresses Discrimination” confrontation if the participant addressed the act of discrimination by speaking up or doing something in response. Detailed definitions are located in Supplementary Tables S1–S7. Illustrative excerpts for these codes are highlighted in Descriptions 1 and 2 below. Figure 5 presents the frequency with which these different codes were applied. The graph shows that roughly 38% of the sample chose to “Avoid Confrontation/Avoid Escalation.” In contrast, an estimated 47% of participants reported “Confront/Directly Addresses Discrimination.”

1. Descriptions of Avoiding or Suppressing Emotion

For the “Avoiding Confrontation/Emotional Suppression” code, we combined “Avoiding Confrontation/Avoid Escalation” and “Inward Suppression of Emotion” because of conceptual and empirical overlap (35). Broadly, these lived experience descriptions were classified as “Avoidance or Suppression” if participants were aware of their distress and the injustice but chose to avoid confrontation and did not engage with the perpetrator (Excerpts B.1.1 and B.1.2) or if they suppressed the expression of their own emotions and responses following the act of discrimination (Excerpts B.1.3 and B.1.4).

B.1.1. “I just had to ignore her and walk away because I did not want to go to jail.” (Woman, 20–30).

B.1.2. “It made me feel angry, but I did not portray that; of course upset, but I did not say much. Because it was at work, I tried to stay professional.” (Woman, 40–50).

B.1.3. “I shut down, and refused to talk to him, or to anyone.” (Woman, 30–40).

B.1.4. “So, and I felt really bad about that [The participant’s teacher told him that he would never be able to join the military with his grades]; it made me feel, it made me feel like I could not do anything after that when he told me that. So I kind of just like, I did not say anything or do anything about it, I just kind of like kept it in as I grew up.” (Man, 20–30).

2: Descriptions of Confronting or Directly Addressing Discrimination

These lived experience descriptions were classified as “Confronts/Directly Addresses Discrimination” if the participant directly identifies the maltreatment as discrimination (Excerpt B.2.1), asks for an apology (B.2.2), or persists in communicating with the perpetrators to complete their goals despite the discriminatory behavior (Excerpt B.2.3).

B.2.1. “[The participant was filling out his timesheet at work] This Caucasian gentleman he walked up to me, my supervisor at that, and he said to me, he asked me, he said, “What are you doing? Are you signing over the deed to the reservation?” I was shocked…[Participant discussed the issue with other supervisors, but they claimed it was a dead issue because he waited too long to report it]… I expressed to him that what he said is inappropriate and wrong, and he should not have said that. He claimed he wasn’t trying to offend me, it was a joke. He said it in a joking manner. But how you could joke with someone you never even joked with before?” (Man, 40–50).

B.2.2 In response to the incident described in A.2.1 (Following an episode in which the participant was asked to translate material as the manager assumed she was Hispanic). The participant went to the manager and asked for an apology, which she received.

B.2.3 (Initial incident is described in excerpt A.1.4) I said, “But I am. I may look different, but I am.” He said, “Well, what do you want to know?” I says, “Well, my tribe is [tribe name],” I said, “and who do I talk to about learning about my people?” (Man, 50–60).

### 3.5 C: Descriptions of resilience

This lived experience description was applied when participants reported ways in which they were resilient. Resilience was identified when individuals reported they were able to effectively manage discriminatory behavior or their emotional reaction to the discrimination. Often, they described reframing the experience or their reactions to the experience (Excerpt C.1-C.3); reported they experienced personal growth because of the way they handled it (Excerpt C.4), or valued their ability to lead others and themselves to change their perceptions (Excerpt C.5).

C.1. “…I did not like it, but, you know, I was only one person. What could I do, you know. But I did not let it, you know, feed me that I was that person, you know, because I knew I wasn’t you know. I knew I wasn’t lazy. I knew I wasn’t you know, dirty. I knew I wasn’t uneducated, you know. So, you know, I did not let that affect me that much. It did affect me when, you know, it was happening, but like I said, I think as people get older and you, you know, observe, analyze and all that stuff, you know, you’ll find yourself a better outcome than, you know, trying to be hateful back. You know, cause it do not solve nothing. And I wonder if, you know, those people in those surrounding towns ever understood that. You know, what is it going to resolve. You know, it just escalates and increases hatred, you know, treating people bad, you know.” (Man, 40–50).

C.2. “Now that I’m older I’m able to cope with it more and it does not really phase me… Like, just degrading stuff, like, it do not really get to me anymore because I know, like, how it is, and how my family is and stuff like that.” (Man, 20–30).

C.3. “There’s not really much I can say or do. I just continue to pray for them, pray for peace in their heart to get their hate out for being judgmental against a person of a darker skin complexion when we all just pump the same blood. We all have one heartbeat, two eyes, one mouth, one nose. We’re all human” (Man, 30–40).

C.4. “You tend to ask why a lot because, you know, it (rejection) does not feel good. But when you find something that does make you feel good and something that you can identify with that other people cannot, then it takes away the bad feeling and replaces it with more of a good feeling, and that feeling is more like acceptance, you know. You guys might not accept me and that’s because you do not understand, but I’m different and I have to accept me because you cannot understand because you are not me. So it just gave me an identity that I wasn’t being given at home.” (Man, 30–40).

C.5 (continued from excerpt B.2.3) He escorted me over to a counselor. We sat down. We talked. But the feeling that I got was I wasn’t perceived as Native American, because they seen the natural, my skin color, and just the overall appearance. But after a while, they got comfortable about being around me, and they started asking me questions. So, that ensued, I get to ask them questions. So, I start asking them about what tribe they are from and how they came to the community center, and it made me feel a little bit at ease. And by me talking to them and them talking -- and I’m talking back and forth, they got comfortable with me, and so they learned a little bit about me and my family history, and that’s it. It just -- it was a different experience. It made me perceive people different, not because of the way they look or they dress, it’s just by talking to them. And I feel, you know, I feel a little empowered.” (Man, 50–60).

## 4 Discussion

To provide a framework for understanding the types of discriminatory experiences facing AI/AN People, we examined the quantitative data and provided qualitative accounts of these experiences in the participants’ own words. These first-hand accounts can help build understanding and awareness for others to remember the types of maltreatment AI/AN People face in the U.S. Their lived experiences can provide an affective context, potentially motivating individuals to act to prevent and ultimately greatly reduce discrimination toward the First Peoples of North America.

The episodes of maltreatment presented by the participants were consistent with existing models of discrimination. Both social threats and physical threats were reported, with social threats more common (Figure 5). Example excerpts were provided of subtypes of social threat, including social exclusion, stigmatization, and discrimination. Many of the social threats experienced by AI/AN People conform to common stereotypes about underrepresented groups, consistent with the notion of stigmatization. These are all stereotypes that stigmatize AI/AN People and communicate that they are “other,” less acceptable, and more dangerous than members of the majority group. These stereotypes include the ideas that AI/AN People are unclean, dangerous/savage, or alcoholic (17, 36). In the examples we included, participants were treated as if they were unclean (e.g., asked them to get out of the pool – Excerpt A.1.2.); dangerous (e.g., told them they would need to go to jail for the rest of their life – Excerpt A.4.3.); or savage (e.g., mimicking examples of Native calls as represented in movies – Excerpt A.3.3.). These stereotypes have been used to justify social and physical threat (17). As these examples demonstrate, negative stereotypes are translated into interpersonal maltreatment across a variety of contexts.

More specific to AI/AN groups are communications of invisibility both on an individual and a population level (18, 20, 37, 38). The communications of invisibility take several forms. In some cases, discriminatory treatment reflects expectations others have of AI/AN culture and AI/AN-specific phenotypes. For example, a perpetrator may view an AI/AN person as from another culture/ethnicity or declare their phenotype or name as inconsistent with their expectations about members of an Indigenous community. For two of the participants, a cultural attribute, such as long hair, is labeled as a problem with gender or sexual orientation rather than recognized as a marker of intelligence or strong Indigenous cultural identity (Excerpt A.1.5. and Excerpt A.1.6.). In other cases, invisibility is communicated through omission, when political leaders or news stories omit mention of AI/AN Peoples or their concerns (Excerpt A.1.7).

AI/AN People have also been stereotyped as less competent and lazy (17). Our participants provided examples of mistreatment consistent with these stereotypes, including experiences about others refusing to allow an AI/AN individual to treat a patient – Excerpt A.2.2. or asking if an AI/AN participant “will sign away the reservation” when completing forms at work– Excerpt A.3.1. The stereotypes communicated can also be considered within the Stereotype Content Model proposed by Cuddy and Fiske (39). This model suggests that stereotypes can be organized along two primary dimensions: warmth and competence. The negative stereotypes, including those associated with dangerousness and invisibility are associated with low warmth. The acts of workplace discrimination and stigmatization are consistent with notions of low competence.

Perpetuation of these and other stereotypes can have significant consequences. These negative stereotypes and those associated with invisibility and denial of culture may have emerged from the need to justify stealing Indigenous lands (39–42). The narrative that AI/AN People were savages and had no civilized culture of their own further justified this theft of tribal lands (17). Stereotypes about criminality or irresponsibility may drive the high rates of arrest and incarceration to which AI/AN People are subject (43).

Consistent with other qualitative studies, the participants’ experiences coping with discrimination by avoiding or confronting the perpetrators provide insight into the human toll of exposure to discrimination. Participants expressed concerns about the interpersonal or professional costs of communicating their anger (including, examples where people feared expressing their concerns because they may go to jail or would cause harm) (See Excerpts A.3.4, B.1.1). Participants also discussed the personal costs of the failure to express their distress, describing how upsetting it was to not communicate how they felt in the moment (See Excerpt B.1.4). Yet other participants provide models of ways to clarify and communicate, by asking for apologies and correcting assumptions (See Excerpts B.2.1, B.2.2, B.2.3, C.5). They frame their coping as a form of resilience, drawing pride from their culture and Tribal Nations. They show their ability to endure and address hardship and even have empathy for those that commit acts again them (See Excerpts C.1 – C.5). Research has demonstrated the associations of discrimination and race related stress to poor health outcomes (44). The description of the participants’ mental processes, as they describe their experiences and coping strategies, provides insights into the pathways through which discrimination can take a toll on physical and mental health. These findings and related data engender a call to action in the public health arena to educate the public about the types of discriminatory challenges facing the AI/AN community and support efforts to prevent discrimination and increase resilience.

There are several limitations to this study. Although we received qualitative data from a large number of AI/AN participants (*n* = 294), the data were collected from a group in the Denver-metro area; therefore, the results will not be similar to other Tribal groups living on reservation-based or rural areas and certainly does not reflect other urban Indigenous community’s experiences. We used content analysis as a method to match to pre-existing structures, but did not probe or ask for clarification of the participants’ experiences of discrimination. The data were collected in the context of a laboratory protocol asking for experiences of discrimination. The structured prompts for disclosure of discriminatory experiences may have limited the nature of the experiences communicated. However, the content of the laboratory prompts and the direction of the research were reviewed by the community advisory board from the time of study design to the end of data collection to ensure accuracy of experiences, understanding of the context, and to correct any assumptions researchers may have made in analyzing the data. In addition, the research assistants who coded participant excerpts were not AI/AN individuals, which may have resulted in some nuances of the participants’ experiences being missed. However, five of the six coders were themselves members of other underrepresented groups (i.e., Black, Latino/a, or Asian). All coders had been participating in a research program investigating experiences of racial discrimination and their effects on health for at least 1 year.

## 5 Conclusion

Experiences of discrimination can take many forms and can be challenging to interpret. When these experiences are organized within standard models of discrimination, individuals may more readily recognize acts of discrimination and may be able to identify experiences of discrimination. This recognition can further facilitate the development of educational and public health interventions to prevent discrimination and mitigate its effects on the health and well-being of American Indian or Alaska Native People. In addition, those who are non-Indigenous may be able to become more effective allies and potentially reduce inadvertent communication of prejudice.

## Data Availability

The raw data supporting the conclusions of this article will be made available by the authors, without undue reservation.

## References

[1] Bui AL Coates MM Matthay EC. Years of life lost due to encounters with law enforcement in the USA, 2015–2016. J Epidemiol Community Health. (2018) 72:715–8. doi: 10.1136/jech-2017-210059, PMID: 29735570

[2] Blair IV Danyluck C Judd CM Manson SM Laudenslager ML Daugherty SL. Validation of the brief perceived ethnic discrimination questionnaire–community version in American Indians. Cult Divers Ethn Minor Psychol. (2021) 27:47. doi: 10.1037/cdp0000419, PMID: 32804521

[3] Gone JP. Redressing first nations historical trauma: theorizing mechanisms for indigenous culture as mental health treatment. Transcult Psychiatry. (2013) 50:683–706. doi: 10.1177/1363461513487669, PMID: 23715822

[4] Krieger N. Embodying inequality: a review of concepts, measures, and methods for studying health consequences of discrimination. Int J Health Serv. (1999) 29:295352. doi: 10.2190/M11W-VWXE-KQM9-G97Q, PMID: 10379455

[5] Williams DR Mohammed SA. Discrimination and racial disparities in health: evidence and needed research. J Behav Med. (2009) 32:20–47. doi: 10.1007/s10865-008-9185-0, PMID: 19030981 PMC2821669

[6] Jones ML. Galliher RV. Daily racial microaggressions and ethnic identification among Native American young adults. Cultural Diversity and Ethnic Minority Psychology. (2015) 21:1 25090153 10.1037/a0037537

[7] D’Amico EJ Dickerson DL Brown RA Klein DJ Agniel D Johnson C. Unveiling an ‘invisible population’: health, substance use, sexual behavior, culture, and discrimination among urban American Indian/Alaska native adolescents in California. Ethn Health. (2021) 26:845–62. doi: 10.1080/13557858.2018.1562054, PMID: 30626198 PMC7510334

[8] Whitbeck LB Hoyt DR McMorris BJ Chen X Stubben JD. Perceived discrimination, and early substance abuse among American Indian Children. J Health Soc Behav. (2001) 42:405–24. doi: 10.2307/3090187, PMID: 11831140

[9] Brown RA Dickerson DL Klein DJ Agniel D Johnson CL D’Amico EJ. Identifying as American Indian/Alaska Native in urban areas: Implications for adolescent behavioral health and well-being. Youth and Society. (2021) 53, 54–75. 34176991 10.1177/0044118x19840048 PMC8232344

[10] Dickerson DL Brown RA Klein DJ Agniel D Johnson C D’Amico EJ. Overt perceived discrimination and racial microaggressions and their association with health risk behaviors among a sample of urban American Indian/Alaska Native adolescents. Journal of Racial and Ethnic Health Disparities. (2019) 6, 733–742. doi: 10.1007/s40615-019-00572-1 30788812 PMC6661006

[11] Johansson P Jacobsen C Buchwald D. Perceived discrimination in health care among American Indians/Alaska natives. Ethn Dis. (2006) 16:766–71. PMID: 17061725

[12] Findling MG Casey LS Fryberg SA Hafner S Blendon RJ Benson JM. Discrimination in the United States: experiences of native Americans. Health Serv Res. (2019) 54:1431–41. doi: 10.1111/1475-6773.13224, PMID: 31657013 PMC6864378

[13] Keeys M Baca J Maybank A. Focus: preventive medicine: race, racism, and the policy of 21st century medicine. Yale J Biol Med. (2021) 94:153. PMID: 33795992 PMC7995954

[14] Ozier EM Taylor VJ Murphy MC. The cognitive effects of experiencing and observing subtle racial discrimination. J Soc Issues. (2019) 75:1087–115. doi: 10.1111/josi.12349

[15] Brondolo E Kelly KP Coakley V Gordon T Thompson S Levy E. The perceived ethnic discrimination questionnaire: development and preliminary validation of a community version 1. J Appl Soc Psychol. (2005) 35:335–65. doi: 10.1111/j.1559-1816.2005.tb02124.x, PMID: 27885969

[16] Contrada RJ Ashmore RD Gary ML Coups E Egeth JD Sewell A. Ethnicity-related sources of stress and their effects on well-being. Curr Dir Psychol Sci. (2000) 9:136–9. doi: 10.1111/1467-8721.00078, PMID: 39018485

[17] Robertson DL. Invisibility in the color-blind era: examining legitimized racism against indigenous peoples. Am. Indian Quart. (2015) 39:113–53. doi: 10.5250/amerindiquar.39.2.0113

[18] House LE Stiffman AR Brown E. Unraveling cultural threads: a qualitative study of culture and ethnic identity among urban southwestern American Indian youth parents and elders. J Child Fam Stud. (2006) 15:393–407. doi: 10.1007/s10826-006-9038-9

[19] Contrada RJ Ashmore RD Gary ML Coups E Egeth JD Sewell A. Measures of ethnicity-related stress: psychometric properties, ethnic group differences, and associations with well-being 1. J Appl Soc Psychol. (2001) 31:1775–820. doi: 10.1111/j.1559-1816.2001.tb00205.x

[20] Juntunen CL Barraclough DJ Broneck CL Seibel GA Winrow SA Morin PM. American Indian perspectives on the career journey. J Couns Psychol. (2001) 48:274–85. doi: 10.1037/0022-0167.48.3.274

[21] Skewes M. Blume A. (2019). Understanding the link between racial trauma and substance use among American Indians. Available at: https://psycnet.apa.org/fulltext/2019-01033-008.html 10.1037/amp0000331 PMC6338088 30652902

[22] Silva EO Gillmann CJ Tate KL. (2018). Confronting institutional discrimination in a color-blind world. Qualitative Sociology Review, 14, 84–108. doi: 10.18778/1733-8077.14.1.05, PMID: 10513145

[23] Brown RA Dickerson DL D’Amico EJ. Cultural identity among urban American Indian/Alaska native youth: implications for alcohol and drug use. Prev Sci. (2016) 17:852–61. doi: 10.1007/s11121-016-0680-1, PMID: 27450682 PMC5030149

[24] Solomon TGA Starks RRB Attakai A Molina F Cordova-Marks F Kahn-John M. The generational impact of racism on health: voices from American Indian communities: study examines the generational impact of racism on the health of American Indian communities and people. Health Aff. (2022) 41:281–8. doi: 10.1377/hlthaff.2021.01419, PMID: 35130067

[25] Leveque D. M. (1994). Cultural and parental influences on achievement among native American students in Barstow unified School District. Paper presented at the national meeting of the Comparative and International Educational Society, San Diego, CA.

[26] Montgomery D Miville ML Winterowd C Jeffries B Baysden MF. American Indian college students: an exploration into resiliency factors revealed through personal stories. Cult Divers Ethn Minor Psychol. (2000) 6:387–98. doi: 10.1037/1099-9809.6.4.387, PMID: 11089314

[27] Krieger N. Racial and gender discrimination: risk factors for high blood pressure?. Social science and medicine. (1990) 30:1273–1281. doi: 10.1093/oxfordjournals.aje.a115571 2367873

[28] Whitbeck LB McMorris BJ Hoyt DR Stubben JD LaFromboise T. Perceived discrimination, traditional practices, and depressive symptoms among American Indians in the upper Midwest. J Health Soc Behav. (2002) 43:400–18. doi: 10.2307/3090234, PMID: 12664673

[29] Willeto AA Sanderson PR Barger SD Teufel-Shone NI. “If you’re down, you know, get up, be proud of yourself, go forward”: exploring urban southwest American Indian individual resilience. Am Indian Alaska Native Mental Health Res. (2023) 30:53–81. doi: 10.5820/aian.3001.2023.53 37027500

[30] Masten AS. Ordinary magic: resilience processes in development. Am Psychol. (2001) 56:227. doi: 10.1037/0003-066X.56.3.227, PMID: 11315249

[31] Danyluck C Blair IV Manson SM Laudenslager ML Daugherty SL Jiang L. Older and wiser? Age moderates the association between discrimination and depressive symptoms in American Indians and Alaska Natives. J Aging Health. (2021) 33:10S–7S. doi: 10.1177/08982643211013699, PMID: 34167343 PMC9087640

[32] Danyluck C Blair IV Manson SM Laudenslager ML Daugherty SL Brondolo E. Discrimination and sleep impairment in American Indians and Alaska natives. Ann Behav Med. (2022) 56:969–76. doi: 10.1093/abm/kaab097, PMID: 34864832

[33] Spielberger CD Reheiser EC. Assessment of emotions: anxiety, anger, depression, and curiosity. Appl Psychol Health Well Being. (2009) 1:271–302. doi: 10.1111/j.1758-0854.2009.01017.x

[34] Krieger N. Discrimination and health. Soc Epidemiol. (2014) 1:36–75. doi: 10.2190/HS.44.4.b

[35] Peters BJ Overall NC Cameron LD Hammond MD Low RS Girme YU. Do habitual emotional suppression measures predict response-focused situational suppression during social interactions? Emotion. (2020) 20:1005. doi: 10.1037/emo0000620, PMID: 31192664

[36] Burkley M Burkley E Andrade A Bell AC. Symbols of pride or prejudice? Examining the impact of native American sports mascots on stereotype application. J Soc Psychol. (2017) 157:223–35. doi: 10.1080/00224545.2016.1208142, PMID: 27383071

[37] Carter RT Forsyth J. Reactions to racial discrimination: emotional stress and helpseeking behaviors. Psychol Trauma Theory Res Pract Policy. (2010) 2:183–91. doi: 10.1037/a0020102

[38] Langer CL Furman R. Exploring identity and assimilation: research and interpretive poems. Forum Qual Soc Res. (2004) 5:2. doi: 10.17169/fqs-5.2.609

[39] Fiske ST Cuddy AJC Glick P Xu J. A model of (often mixed) stereotype content: competence and warmth respectively follow from perceived status and competition. J Pers Soc Psychol. (2002) 82:878–902. doi: 10.1037/0022-3514.82.6.878, PMID: 12051578

[40] Cuddy AJC Fiske ST Glick P. The BIAS map: behaviors from intergroup affect and stereotypes. J Pers Soc Psychol. (2007) 92:631–48. doi: 10.1037/0022-3514.92.4.631, PMID: 17469949

[41] Fiske ST Cuddy AJC Glick P. Universal dimensions of social cognition: warmth and competence. Trends Cogn Sci. (2007) 11:77–83. doi: 10.1016/j.tics.2006.11.005, PMID: 17188552

[42] Fiske ST Xu J Cuddy AC Glick P. (dis)respecting versus (dis)liking: status and interdependence predict ambivalent stereotypes of competence and warmth. J Soc Issues. (1999) 55:473–89. doi: 10.1111/0022-4537.00128

[43] Tighe S. ‘Of course we are crazy’: discrimination of native American Indians through. Crim Justice, Justice Policy Journal. (2014) 11:10–11.

[44] Brondolo E Blair IV Kaur A. Biopsychosocial mechanisms linking discrimination to health: a focus on social cognition In: Major B Dovidio JF Link BG, editors. The Oxford Handbook of stigma, discrimination, and health, vol. 1. New York, NY: Oxford University Press (2018)

[45] Brondolo E Kaur A Flores M. Structural racism and health in the age of COVID-19: a selective review with policy implications. Soc Issues Policy Rev. (2023) 17:34–61. doi: 10.1111/sipr.12095

[46] Brondolo E Thompson S Brady N Appel R Cassells A Tobin JN. The relationship of racism to appraisals and coping in a community sample. Ethn Dis. (2005) 15:14–9. 16315377

[47] Colorado Commission of Indian Affairs. Available at: https://ccia.colorado.gov/ (Accessed May 5, 2021).

[48] Norris T Vines PL Hoeffel EM. The american indian and Alaska native population: 2010. Washington, DC: US Department of Commerce, Economics and Statistics Administration, US Census Bureau (2023).

