# Supplementary material for: Discrimination among American Indian and Alaska Native people: implications for public health communication
Source: Front Public Health. 2024 Oct 22;12:1384608. doi: 10.3389/fpubh.2024.1384608 (PMC11537154; doi:10.3389/fpubh.2024.1384608)
Supplement: Supplementary file 1 [file Data_Sheet_1.docx]

Supplementary Material

# Supplementary Data

# Supplementary Figures and Tables

## Supplementary Tables

**Detailed Definitions of Codes/Variables**

The following distinct codes were used to classify the elements of the experiences of discrimination that research participants discussed:

**Table 1. Act of Discrimination:** *This category of codes refers to what the speaker has categorized as prejudice or unjust treatment.*

| Social Threat: This code was used when the participant discussed being left out or ignored. This also included labels or statements that indicate a negative attitude or stereotypes. In addition, this applied to any time the participant felt treated unfairly or harassed (e.g., they were followed, detained, threatened, questioned, profiled, falsely accused, etc.). This code does NOT incorporate physical threats. |
| --- |
| Physical Threat: This code was applied to acts of discrimination that were physically violent, aggressive, or threatening. |
| Other: This code is appropriate when the act of discrimination did not obviously fit into any other category. In other words, it was used when the discriminatory situation was unclear as being physical, verbal, or both. |

**Table 2. First Party (Speaker) Response/Intent**: *This section relates to the speaker’s immediate reaction to the act of discrimination.*

| Avoid Confrontation/Avoid Escalation: This indicates that the speaker chose to remove themselves from the situation or disengage with the offender (e.g., uses phrases like “I walked away”, “I told others that it’s no big deal, just leave it”, “I didn’t correct them”, “I was mad, but I laughed along”). |
| --- |
| Spoke to Authority: This code points out that the speaker went to an authority figure (e.g., teacher, boss, law enforcement, etc.) to talk about the act of discrimination or report the perpetrator. |
| Didn’t Know What to Do: This was assigned when the speaker stated that they did not know how to respond to or feel about the discrimination that they faced. Specifically, it was used to refer to actions. |
| Confront/Directly Addresses Discrimination: This was administered when the speaker addressed the act of discrimination by speaking up or doing something in response to it (e.g., “I asked the cashier why they thought I couldn’t pay the bill” or “I called him a jerk”). |
| Identity Concealed or Disclosed: This code was applied when the speaker hid or revealed their racial identity to the perpetrator of the act of discrimination. Actions that were classified as the speaker concealing their racial identity could include endeavors like covering up hair, pretending to be a different race/ethnicity, removing regalia, etc. |
| Inward Reflection of Negative Emotions: This was used when the speaker realizes their negative emotions and tries to control them through factors like breathing and counting to prevent a negative emotional reaction to the act of discrimination (e.g., “I took a deep breath” or “I counted to 20”). |
| Inward Suppression of Negative Emotions: This describes when the speaker recognized their negative emotions and decided to shut down or hold their tongue to prevent a negative emotional reaction to the act of discrimination (e.g., “I held it in” or “I shut down”). |
| Biological Response Symptoms: This refers to the speaker mentioning that they experienced physiological responses to the discrimination (e.g., heart-pounding, light-headedness, feeling sick, vomiting, got a headache, clenched fists, sweating palms). |
| Psychological Symptoms: This code was applied when the speaker reported that they were affected by persistent or extreme “psychological” responses after the discriminatory event occurred (e.g., nightmares, panic attacks, persistent distress, anxiety, depression, rumination, etc.). |
| Redirect Attention/Move Past: This was administered when the speaker mentioned the need to move past the experience (i.e., “I need to let it go” or “My mother told me not to let it bug me”). |
| Indirect Action to Cope: This code was employed when the speaker took up a behavior like smoking, drinking, or eating in direct response to the act of discrimination. |

**Table 3. First Person Speaker Emotions/Emotional Coping**: *This division of codes describes when the speaker indicated that they felt an emotion or used a coping mechanism in response to the act of discrimination.*

| Negative – Fight Approach: This code signifies that the speaker stated they felt angry, hostile, mad, irritated, pissed off, etc. in response to the discrimination. |
| --- |
| Negative – Avoid/Flight: This code illustrates that the speaker expressed that they felt fearful, upset, nervous, sad, miserable, embarrassed, disgusted, etc. in response to the discrimination. |
| Positive: This was applied when the speaker mentioned that they felt proud, happy, relieved or content. |
| Surprise/Shock/Stunned/Amazed: This refers to the speaker disclosing that they didn’t know how to feel, or saying that they felt surprised, shocked, stunned, or amazed in response to the act of discrimination. |

**Table 4. Location of Discrimination**: *This category specifies where the discriminatory incident occurred.*

| In Public: This code was used when the discriminatory event took place in a public setting (e.g., store, restaurant, bus stop, street) |
| --- |
| Other Institution: This refers to an act of discrimination that occurred in any institution that is funded by the government (e.g., bank, university, military, court/parole office, hospitals/medical offices, etc.). It was also used if the location was online, and as a catch-all if the location wasn’t explicitly stated. |
| Private Residence: This code was used when the speaker articulated that the act of discrimination happened in a home. |
| School/Work: This was assigned when the location of the act of discrimination was an educational setting or place of work. |

**Table 5. Offender Identity**: *This section of codes relates to the person who is identified by the speaker as the perpetrator of the discriminatory incident.*

| Family Member: This was used when the perpetrator was a biological or adopted family member. |
| --- |
| Peer/Friend: This was applied when the perpetrator was a person who the victim shared a similar age or status with (e.g., co-worker, classmate, friend). |
| Service Provider: This code was employed when the perpetrator was a service provider, such as a clerk, cashier, banker, nurse, landlord, etc. |
| Teacher/Boss/Law Enforcement: This code was assigned when the perpetrator was a person that had direct authority over the victim. |
| Stranger/Unknown: This was used when the perpetrator was somebody that the speaker does not know, or the speaker didn’t specifically identify the perpetrator. |

**Table 6. Third Party Response**: This division of codes describes the bystander’s reaction to the discrimination or perpetrator, or the speaker’s reaction to a discriminatory incident where they were not the target.

| Negative (invalidates or escalates): This code was applied when the speaker believed that a third party made the situation worse. |
| --- |
| None: This was used when the speaker stated that a third party made no response to the act of discrimination. |
| Positive (support/validate): This code was assigned when the speaker believed that a third party made the situation better. |

**Table 7. Other:** Categories that did not fit into a major theme

| AI-AI discrimination: This was implemented when the speaker specified that the perpetrator of the act of discrimination was another American Indian/Alaska Native. |
| --- |
| Attributional Ambiguity: This code was utilized when the speaker mentioned any uncertainty regarding whether the act of discrimination occurred due to their race. |
| Coping After Situation: This was used when the speaker expressed that they coped with the situation after the incident had transpired (e.g., “Later when I got home, I ate and watched my favorite tv show to get my mind off things”. |
| First Person Speaker with Someone: This code was applied when the speaker mentioned that they were accompanied by someone they knew when the act of discrimination took place. This didn’t solely refer to anyone that may have been on the scene. |
| First Person Speaker Interpersonal Perceptions: This describes how the speaker felt in relation to the perpetrator or other people in general. It also covered when the speaker felt and communicated feelings of being shut out, invalidated, excluded, rejected, ignored, invisible or discounted, distrusted, harassed, judged, targeted, or singled out. |
| Indirect/Vicarious Discrimination: This refers to the target of the discrimination not being the speaker. |
| Offender Response/Persists: This code indicates that the perpetrator of the act of discrimination was confronted and had a response to the confrontation (e.g., acknowledges harm, denial, or anger). |
| Reference of Race/Ethnicity: This was applied when the speaker referenced their own or another person’s race/ethnicity. |
| Resilience: This was appointed when the speaker mentioned the process of adapting well in the face of discrimination. This also refers to the speaker mentioning “bouncing back” from the discriminatory experience. |
| Situational Substance Use/Illegal Activity/Violence: This code was selected when the speaker mentioned an unsafe environment or unsafe behavior as part of the setting of their story, and not as part of the offense. |
| Time of Discrimination – Childhood: This code was administered when the act of discrimination occurred during childhood. |

**Detailed Coding Methods**

To prepare the coders for their role in the study, they watched training videos that explained the research that was being conducted, did 2 rounds of practice coding to determine if they were fit to perform the coding up to standard, and discussed the components of the studies further with the research assistants that gathered the data. Also, during the training period, any issues or confusion regarding the code definitions and when the codes should be applied was discussed among the coders, research assistants, and one of the lead researchers, Dr. Brondolo. If necessary, revisions were made to the codes or new codes were produced to describe the data more efficiently. This continued throughout the coding process until the coders were confident in their coding decisions.

Additionally, in order to ensure the inter-rater reliability of the study’s results and that the coders were adequately trained for coding the excerpts, the team coded practice excerpts individually using Dedoose’s Training Center program. To measure the practice results’ inter-rater reliability, the degree to which raters designate the same score to the same variable (McHugh M. L. 2012), the kappa statistic was used. The significance of rater reliability comes from its representation of the degree to which the data gathered in research accurately represents the variables that were measured. Cohen’s Kappa was established to account for the chance that raters guess on, at minimum, some variables as a result of them being uncertain. It has a margin of -1 to +1 like the majority of correlation statistics studies (McHugh M. L. 2012). The coders received Kappas coefficients of agreement ranging from .68-.87, which indicate moderate to high reliability (Viera & Garrett, 2005).

To produce the study’s results and examine the data, we assessed each code’s ratio of excerpts that they appeared in to the total amount of excerpts that were in the study, which was 294. To observe the prevalence of each code’s application, we used the Dedoose code application function to make a chart of the amount of times each code was assigned to an excerpt. Once this was done, we used a 0 to represent a code not being applied to an excerpt and a 1 to indicate that the code was applied. The corresponding percentages of the ratios were calculated in Microsoft Excel by dividing the amount of excerpts each code appeared in by the total amount of excerpts in the study and multiplying them by 100. By analyzing the prevalence of each code’s application this way, we were able to analyze the pervasiveness of each element of discriminatory experiences that we were interested in.

**Table 8. The following table represents the schema that coders used when applying specific codes to passages.**

| **Thought Process** | **Corresponding codes** |
| --- | --- |
| 1. **What happened?** | |
| - 1. When did it happen? | Childhood |
| - 1. What was the type(s) of discrimination? | Physical, Social, Other (unknown type of discrimination) |
| - 1. Where did it happen? | In public, in a private residence, in school (corresponds to childhood) / work |
| - 1. Who were they with? Did they know them? | First person speaker with someone |
| - 1. Did the speaker mention uncertainty about whether the situation was discriminatory or not? | Attributional Ambiguity |
| - 1. Upon confronting or avoiding the offender, did the offender respond (i.e., apologizes, accepts/denies responsibility, acknowledges harm) or persist in discriminating? | Offender Response/Persists |
| - 1. Who was the source of discrimination: AI/AN or other race | Family member, Peer/Friend, Service Provider, Teacher/Boss/Law Enforcement, Stranger; AI-AI Discrimination; Reference to Race/Ethnicity |
| - 1. Was identity concealed: | Identity Concealed/Disclosed (likely corresponds to confront/avoid confrontation) |
| 1. **What did they feel mentally or physically?** | |
| - 1. Type of emotion | Negative Fight, Negative Flight, Positive, Surprise/Shocked/Stunned/Amazed |
| - 1. How was the emotion handled? (suppress, express, turn attention away) | Redirect Attention/Move past, Inward Reflection, Inward suppression |
| - 1. Psychological symptoms, including rumination? | Psychological symptoms |
| - 1. Biological symptoms/bodily response? | Biological Symptoms |
| 1. **How did they or others respond?** | |
| - 1. Did someone outside of the situation witness the discrimination and get involved? How did the speaker (or the person being discriminated) feel about their response? | Negative (invalidates or escalates), None, Positive (support/validate) |
| - 1. Was there any type of response towards the discriminatory act from speaker? | Confront Discrimination, Avoid Confrontation, identity concealed or disclosed, |
| - 1. Was there any type of coping after the discriminatory act? | Indirect Action to Cope, Coping After Situation |
| - 1. Did the individual speak to an authority figure in response to the act? Was there a response? | Spoke to Authority, No Response |
| - 1. Evaluation of future – resilience. Ex. Did the speaker mention that this has happened or happens more than once and choose to respond to it a certain way? | Resilience |
| 1. **Does it change the way they think or act?** | Interpersonal perceptions |
| 1. **Did the speaker mention their home or neighborhood environment being unsafe or with drugs/ alcohol?** | Situational substance use/ illegal activity/violence |

**Table 9. Prevalence and Percent of Themes and Codes**

| **Theme/Codes** | **Prevalence** | **Percent**  **(Number of stories mentioning a code, n=294)** |
| --- | --- | --- |
| **Act of Discrimination** | | |
| Social Threat | 275 | 93.5 |
| Physical Threat | 38 | 12.9 |
| **Behavioral Responses to Discrimination** | | |
| Confront/ Directly Addresses Discrimination | 139 | 47.3 |
| Avoid Confrontation/ Avoid Escalation | 112 | 38.1 |
| **Emotional Responses to Discrimination** | | |
| Inward Suppression of Negative Emotions | 24 | 8.2 |
| Negative - Avoid/ Flight (fear, sadness, embarrassment) | 130 | 44.2 |
| Negative - Fight Approach (anger) | 129 | 43.9 |
| **Social Cognitive Changes** | | |
| Interpersonal Perceptions | 153 | 52.0 |
| **Offender Identity** | | |
| Teacher/Boss/ Law Enforcement | 93 | 31.6 |
| Peer/Friend | 64 | 21.8 |
| Stranger/Unknown Person | 64 | 21.8 |
| Service Provider | 59 | 20.1 |
| Family Member | 18 | 6.1 |
| **Offender's Persistence** | | |
| Offender Response/Persists | 85 | 28.91 |
| **Resilience in Response to Discrimination** | | |
| Resilience | 23 | 7.8 |
